# The efficacy of unsupervised home‐based exercise regimens in comparison to supervised laboratory‐based exercise training upon cardio‐respiratory health facets

**DOI:** 10.14814/phy2.13390

**Published:** 2017-09-15

**Authors:** James Blackwell, Philip J. Atherton, Kenneth Smith, Brett Doleman, John P. Williams, Jonathan N. Lund, Bethan E. Phillips

**Affiliations:** ^1^ MRC‐ARUK Centre for Musculoskeletal Ageing Research University of Nottingham Royal Derby Hospital Centre Derby United Kingdom; ^2^ Division of Surgery and Anaesthetics Royal Derby Hospital Derby United Kingdom

**Keywords:** Blood Pressure, Cardiorespiratory, Exercise, HIIT, HIT

## Abstract

Supervised high‐intensity interval training (HIIT) can rapidly improve cardiorespiratory fitness (CRF). However, the effectiveness of time‐efficient unsupervised home‐based interventions is unknown. Eighteen volunteers completed either: laboratory‐HIIT (L‐HIIT); home‐HIIT (H‐HIIT) or home‐isometric hand‐grip training (H‐IHGT). CRF improved significantly in L‐HIIT and H‐HIIT groups, with blood pressure improvements in the H‐IHGT group only. H‐HIIT offers a practical, time‐efficient exercise mode to improve CRF, away from the laboratory environment. H‐IHGT potentially provides a viable alternative to modify blood pressure in those unable to participate in whole‐body exercise.

## Introduction

The risk of developing cardiovascular (D'Agostino. et al. 2008) and metabolic (Veronica and Esther 2014) disease(s) increases with advancing age. However, aging is not the only risk factor for cardiovascular disease (CVD); sedentary middle‐aged adults have been identified as a specific high‐risk group, with inactive lifestyles associated with all‐cause mortality (Biddle et al. 2010). It therefore follows that exercise is the most well‐established nonpharmacological countermeasure to CVD risk (Myers 2003). Current guidelines state that adults should complete at least 150 min of moderate‐intensity aerobic physical activity throughout the week, or do at least 75 min of vigorous‐intensity aerobic physical per week (WHO 2015). However, less than 40% of men and 30% of women meet these guidelines (UK Department of Health 2011). Poor uptake and adherence to exercise is driven by a multitiude of factors, such as “lack of time,” aversion to exertion, and access to specialist equipment (Trost et al. 2002; Gillen and Gibala 2013). Moreover, these current physical activity guidelines do not consider the potential benefits of novel exercise modes, that is, short intense bouts of exercise, or static isometric training.

High intensity interval training (HIIT) (Trost et al. 2002; Gillen and Gibala 2013) is one such novel exercise mode (Kravitz 2011). Indeed, laboratory‐based (supervised) HIIT (L‐HIIT) has been shown to elicit improvements in cardiorespiratory fitness (CRF), over very short time‐periods (2–6 weeks) in athletes (Iaia et al. 2009), moderately trained (Helgerud et al. 2007; Little et al. 2010), sedentary (Trilk et al. 2011; Klonizakis et al. 2014) and patient groups (Gibala et al. 2012; Weston et al. 2014; Lanzi et al. 2015). These improvements were seen despite low exercise volume and minimal time commitments (Gillen et al. 2014).

Nonetheless, despite these findings supporting the benefits of L‐HIIT, the efficacy of home‐based *unsupervised* HIIT‐based strategies (H‐HIIT), which overcome the need for specialist equipment and personnel, is unknown. Previously most HIIT protocols have been studied in the laboratory setting, however, newer protocols requiring no specialist equipment have been investigated showing positive effects on CRF. Whole body aerobic resistance training (Mcrae et al. 2012) and more recently low volume intense stair climbing (Allison et al. 2017) has improved CRF in untrained females over a 4 week period while supervised but with no specialist equipment. Similarly, while home‐isometric handgrip training (H‐IHGT) is a promising, simple and rapid task that has been shown to lower resting blood pressure (RBP) within ~10 weeks (Millar et al. 2008; Garg et al. 2014), how it compares to HIIT‐based strategies in relation to modulating RBP is unknown. Herein, we aim to resolve this by comparing the effects of H‐HIIT to an already established efficacious supervised L‐HIIT protocol, on *V*O_2_ max and anaerobic threshold (AT). We also aim to compare the effects of H‐IHGT on RBP versus L‐HIIT and H‐HIIT.

## Materials and Methods

### Subjects

Eighteen middle‐aged (52 ± 5 year; 13:5 female:male) individuals (BMI 27.4 ± 3.9 kg/m^2^) not engaged in any formal exercise regime (<2 times per week) were recruited to the study and provided written informed consent (Table 1). Exclusion criteria were as per ATS/ACCP Guideline for CPET (American Thoracic & American College of Chest 2003). Inclusion criteria included no musculoskeletal limitations and availability for the whole study duration. Six subjects were randomly assigned to each intervention group (L‐HIIT, H‐HIIT or H‐IHGT) prior to baseline testing. The study was approved by the University of Nottingham Medical School ethics committee and complied with the Declaration of Helsinki.

### Baseline and post‐training measures

All measurement equipment was calibrated and fully maintained throughout the study period. Subjects' height and weight was measured on arrival. Resting heart rate and noninvasive blood pressure was taken following 5 min seated rest with an automatic blood pressure monitor (A&D Medical, Saitama, Japan) prior to any exercise testing. All subjects then underwent cardiopulmonary exercise testing (CPET; Lode Corival, Lode, Groningen), with inline breath by breath data collected via a metabolic cart (nSpire Zan 600, Germany), using a modified Bruce ramp protocol as previously described (Boereboom et al. 2016). Tests were considered maximal if 3 or more of the following criteria were met: (1) plateau in the oxygen uptake curve (sustained flattening of *V*O_2_ curve despite rising *V*CO_2_); (2) a respiratory exchange ratio (RER) of >1.1; (3) HR over 85% age‐predicted maximum, and (4) a rating of perceived exertion (RPE); modified Borg scale (Borg 1982) ≥9 immediately following the test. CPET interpretation was performed by two independent experienced assessors blinded to time‐point (i.e., pre or post‐training) and group information. *V*O_2_ max values were taken as the highest reading in the last 30 sec of the test. AT was determined using a modified V‐slope and ventilatory equivalents method (Boereboom et al. 2016). All baseline measures were repeated >3 but <7 d after the last training session.

### Training regimes

Volunteers performed their respective regime 3 times each week for 4 weeks. Compliance was monitored via a self‐report training diary (H‐HIIT, H‐IHGT) or attendance (L‐HIIT), and was 100% for each intervention.

**Table 1:** Subject baseline characteristics

	L‐HIIT (*n* = 6)	H‐HIIT (*n* = 6)	H‐IHGT (*n* = 6)
Age (years)	51.5 (2.7)	52.2 (2.0)	51.5 (2.3)
BMI (kg/m^2^)	27.5 (1.1)	26.3 (2.0)	28.3 (1.8)
*V*O_2max_ (mL/kg/min)	26.5 (2.6)	27.8 (1.9)	23.7 (2.4)
*V*O_2AT_ (mL/kg/min)	15.2 (1.1)	13.93 (0.7)	13.6 (1.5)
SBP (mmHg)	127 (6.5)	129 (5.4)	138 (4.2)
DBP (mmHg)	84 (0.9)	81 (5.1)	93 (2.7)

Data depict mean (SD). There were no significant differences between the groups. Analysis via one‐way ANOVA.

L‐HIIT comprised a 2 min unloaded warm‐up, followed by 5 × 1 min exertions at 95–110% of the maximal load (watts (W)) achieved during subjects' baseline CPET (determined by an initial assessment session (Boereboom et al. 2016)), interspersed with 90 seconds unloaded cycling. A 2 min unloaded recovery completed each session. All participants underwent a 10% intensity increase at the mid‐way point of training (after session 6). Participants were given verbal encouragement throughout each session to ensure a rate of cadence sufficient to evoke a HR response greater than 85% predicted maximum (i.e., 220 – age (y)).

H‐HIIT comprised a 2 min jogging warm‐up, followed by 5 × 1 min exertions of three different equipment‐free exercises (star‐jumps, squat thrusts, and static sprints). To try and ensure that exercise intensity remained constant throughout each session, subjects were instructed to complete the maximum number of repetitions possible with good form during each exertion, and to match the number of repetitions achieved during exertions 1 (star‐jumps) and 2 (squat thrusts) during exertions 4 and 5 when these exercises were repeated. Each exertion was interspersed with 90 sec walking, with 2 min light static jogging completing each session.

H‐IHGT comprised 4 × 2 min isometric hand‐grip holds with their dominant hand at 30% of maximal voluntary contraction (MVC), interspersed with 2 min rest periods (Camry EH101 Electronic Hand dynamometer, USA). MVC was recorded as best of three maximal contractions on the dominant arm while stood in the anatomical position (Takei 5401 Grip strength dynamometer, Japan).

### Statistical analysis

Descriptive data are presented as means ± standard deviation. ANCOVA was used to compare postintervention efficacy between groups with preintervention scores as a covariate. Results are presented with Bonferroni adjusted p values. We also tested for the assumption of homogeneity of regression slopes by testing the interaction of the independent variable with the covariate. Paired t‐tests were used for within group analyses. Pearson's correlation was used to test the association between change in blood pressure and baseline values. Statistical significance was set at *P* < 0.05. All analyses were conducted on STATA Version 14.2, SPSS Version 22, and Graphpad Prism Version 6.

## Results

There were no adverse events during the study and all subjects completed all testing and training sessions. All subjects fulfilled our *V*O_2_ max criteria as outlined above. There were no significant differences in body weight (kg) in any group after the training period.

**Figure 1:**
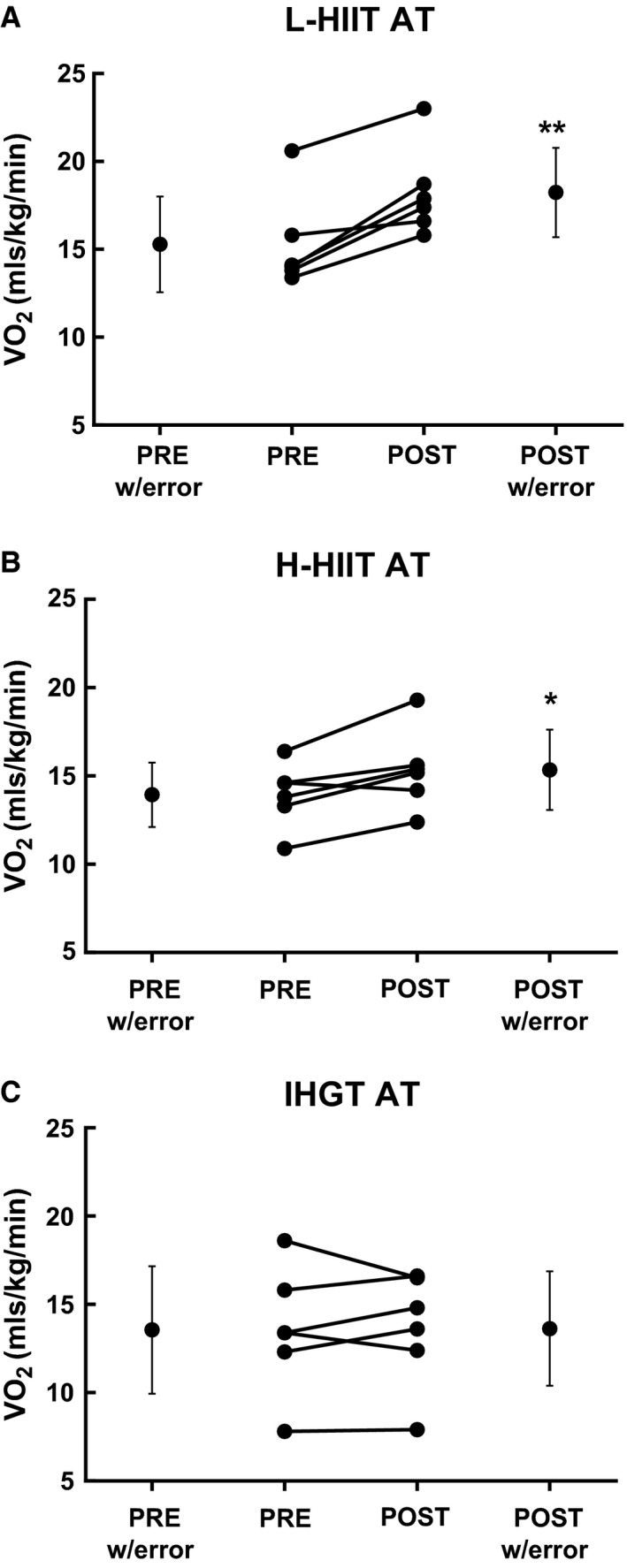
Anaerobic threshold (AT) before (PRE) and after (POST) 4 weeks laboratory‐based high intensity interval training (L‐HIIT; A), home‐based HIIT (H‐HIIT; B) or isometric hand‐grip training (H‐IHGT; C). Graphs depict mean±SD and individual changes. Analysis via paired Students t‐test. *=P < 0.05, **= P < 0.01 versus PRE training.

**Figure 2:**
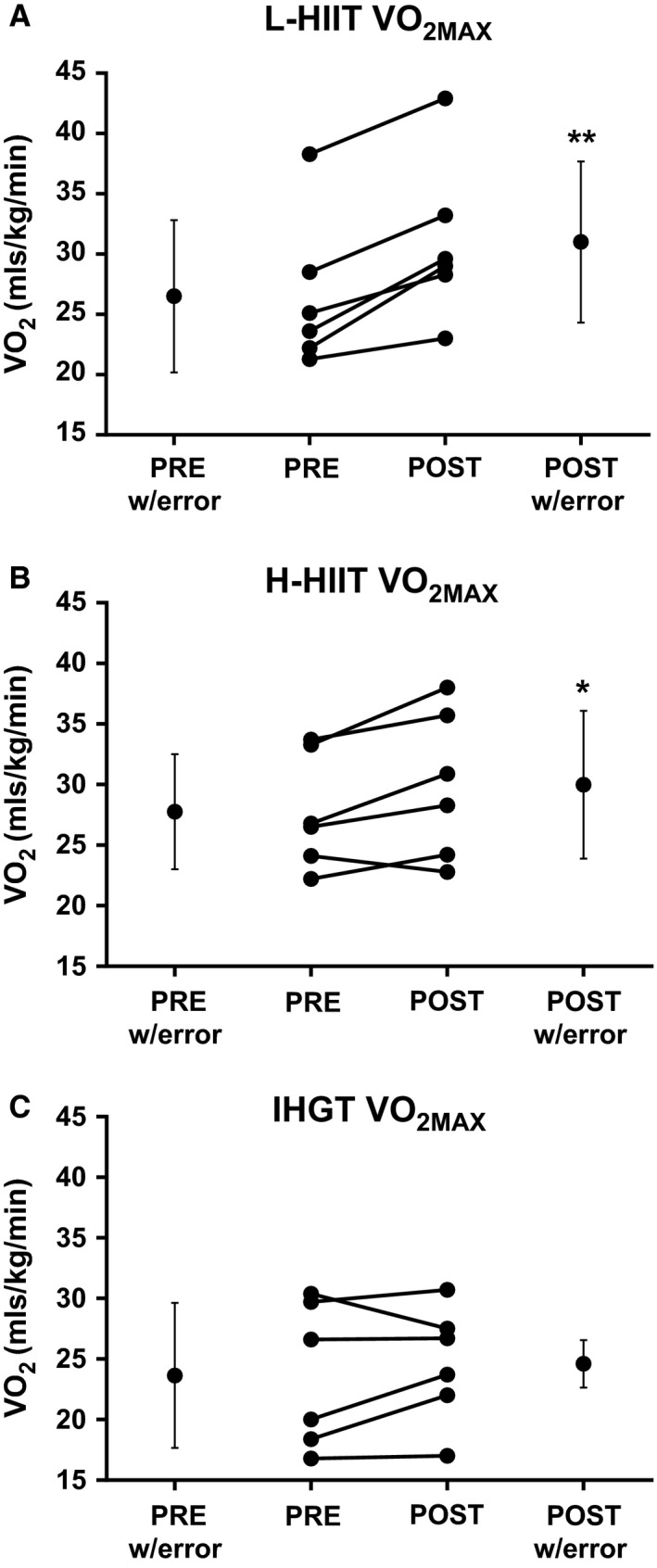
VO_2_max before (PRE) and after (POST) 4 weeks laboratory‐based high intensity interval training (L‐HIIT; A), home‐based HIIT (H‐HIIT; B) or isometric hand‐grip training (H‐IHGT; C). Graphs depict mean±SD and individual changes. Analysis via paired Students t‐test. *= P < 0.05, **= P < 0.01 versus PRE training.

There was a significant mean improvement in CRF in both L‐HIIT (AT: 15.28 ± 2.73 to 18.23 ± 2.54 mL/kg/min, *P* < 0.01; *V*O_2_max: 26.50 ± 6.31 to 31.00 ± 6.69 mL/kg/min, *P* < 0.001) and H‐HIIT (AT: 13.93 ± 1.82 to 15.35 ± 2.27 mL/kg/min, *P* < 0.05; *V*O_2_ max: 27.77 ± 4.75 vs. 29.98 ± 6.094 mL/kg/min, *P *= <0.05), with no significant effect of H‐IHGT (AT: 13.55 ± 3.61 to 13.63 ± 3.25 mL/kg/min, *P* = 0.88; *V*O_2_ max: 23.65 ± 5.98 to 24.60 ± 4.80 mL/kg/min, *P* = 0.39 (Figs. 1 & 2)). L‐HIIT elicited significantly greater improvements in AT and *V*O_2_ max (both *P* < 0.05) when compared with H‐IHGT. There were no other significant differences between the groups. The assumption of homogeneity of regression slopes was not violated (*P* > 0.05 for interaction).

**Figure 3:**
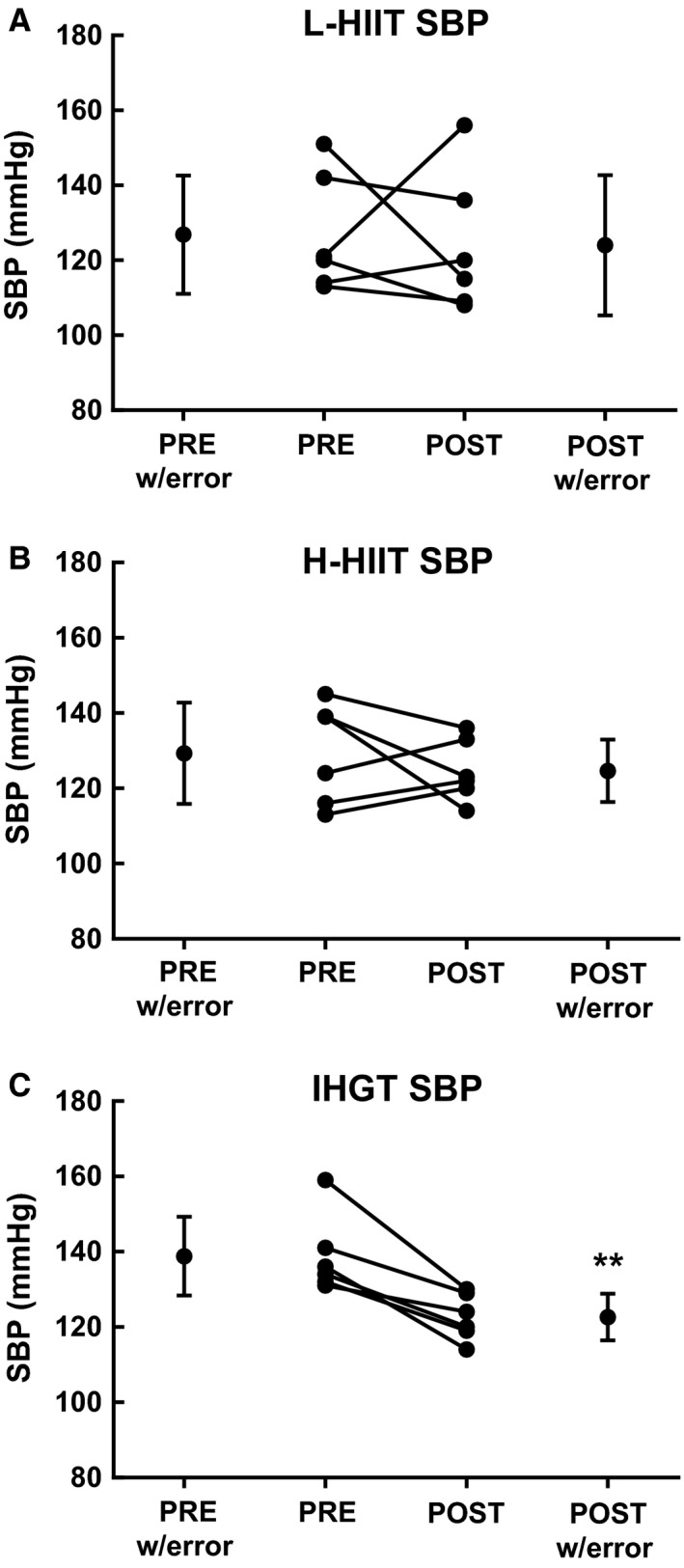
Systolic blood pressure (SBP) before (PRE) and after (POST) 4 weeks laboratory‐based high intensity interval training (L‐HIIT; A), home‐based HIIT (H‐HIIT; B) or isometric hand‐grip training (H‐IHGT; C). Graphs depict mean±SD and individual changes. Analysis via paired Students t‐test. **= P < 0.01 versus PRE training.

**Figure 4:**
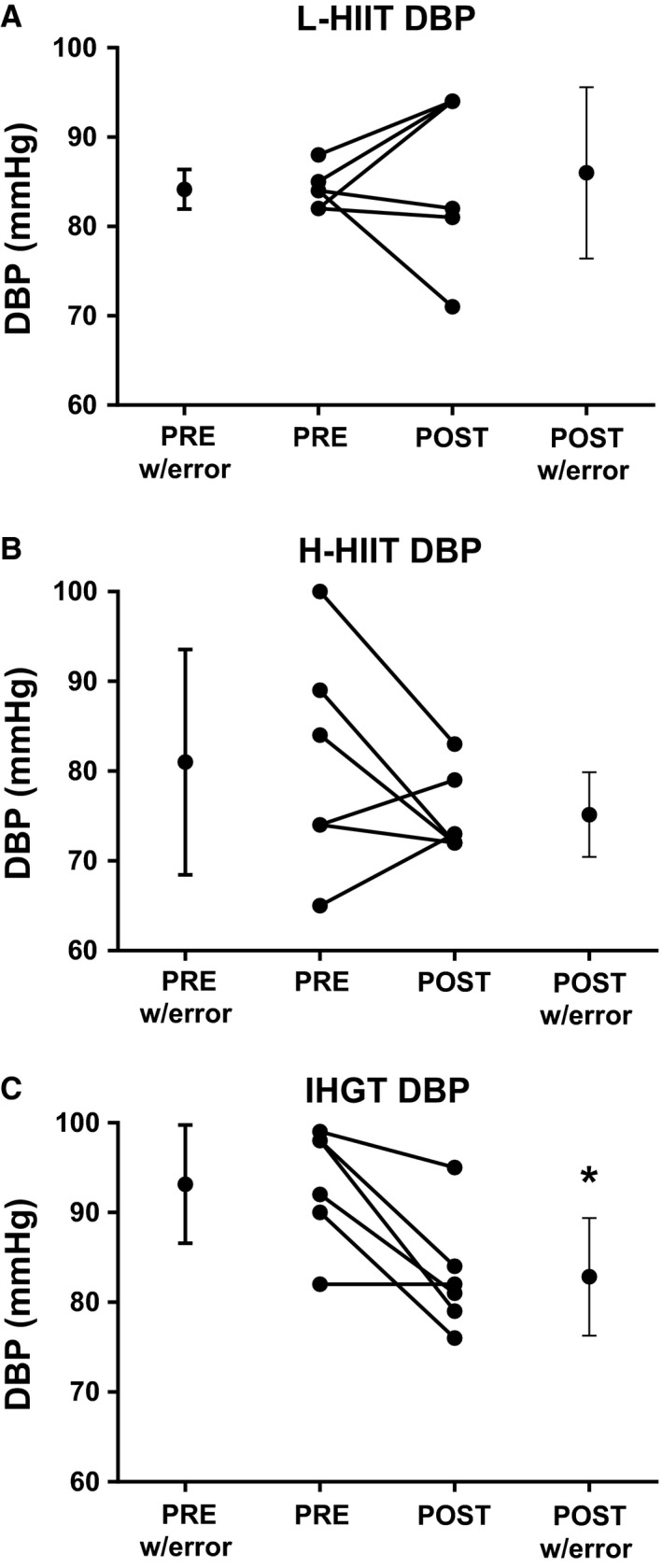
Diastolic blood pressure (DBP) before (PRE) and after (POST) 4 weeks laboratory‐based high intensity interval training (L‐HIIT; A), home‐based HIIT (H‐HIIT; B) or isometric hand‐grip training (H‐IHGT; C). Graphs depict mean±SD and individual changes. Analysis via paired Students t‐test. *= P < 0.05 versus PRE training.

There were no significant differences between the groups' baseline systolic (SBP) or diastolic (DBP) blood pressures. When grouping all subjects together, there was a significant negative correlation between baseline systolic and diastolic blood pressures and change in these values after training (*r* = −0.72; *P* < 0.05 and *r* = −0.64; *P* < 0.05, respectively). SBP (139 ± 4 to 123 ± 3 mmHg, *P* < 0.01) and DBP (93 ± 3 to 82 ± 3 mmHg, *P* < 0.05) decreased significantly in the H‐IHGT group only, with no significant changes in the L‐HIIT or H‐HIIT groups (Figs. 3 & 4).

## Discussion

For the first time, both supervised L‐HIIT and *unsupervised* H‐HIIT have been shown to improve CRF in just 4 weeks using an identical work‐to‐rest ratio. H‐IHGT did not confer benefit in CRF, but did elicit a beneficial effect on SBP in this short 4‐week time frame.

As previously, and consistently shown (Little et al. 2010; Lanzi et al. 2015; Boereboom et al. 2016), L‐HIIT elicited improvements in indices of CRF in just 12 sessions. However, despite this solid and expanding evidence base the mechanistic basis of HIIT‐induced improvements in CRF are not fully elucidated. Increased skeletal muscle mitochondrial capacity (Little et al. 2010) and (central and peripheral) vascular adaptation (Wisløff et al. 2009) have both been postulated to account for improvements in *V*O_2_ max in previous studies, while improvements in muscle buffering capacity (Gibala et al. 2006) and reduced submaximal exercise energy expenditure (Iaia et al. 2009) may account for improvements in AT. Thus, L‐HIIT may represent a time‐efficient method to engage sedentary middle‐aged individuals, identified as at high risk for CVD (Biddle et al. 2010), in a regular physical activity regime with the aim of enhancing aerobic fitness and reducing BP. However, time‐efficacy only combats one of the cited reasons for poor exercise adherence. Indeed, the need for specialist equipment (cycle ergometers) and supervision are notable limitations for this method of training, demanding significant time, and financial commitments.

Interestingly this study demonstrates that *unsupervised* H‐HIIT, without the need for specialist equipment, can also improve CRF in middle‐aged sedentary individuals in just 4 weeks. With an identical time commitment to L‐HIIT, H‐HIIT induced significant gains in both *V*O_2_ max and AT, with no significant difference between the improvements made by these groups. Additionally, H‐HIIT can be easily adapted to account for injury and/or pathologies commonly occurring in middle‐age (e.g., osteoarthritis, urinary stress incontinence), potentially further improving adherence.

To the best of our knowledge the impact of H‐IHGT upon VO_2_ max and other indices of CRF was unknown, here we show no effects in middle‐aged sedentary adults. Perhaps, as would be predicted, in recruiting a significantly smaller muscle mass than both forms of HIIT and offering no significant cardiorespiratory challenge, H‐IHGT did not provide sufficient stimulus to promote improvements in CRF. Nonetheless, H‐IHGT was able to confer significant improvements in resting BP within this cohort. H‐IHGT may provide a viable alternative for those individuals who are unable to participate in dynamic exercise regimes who also have rising blood pressure not yet requiring medical management (accepted hypertension treatment threshold <140/90, (NICE 2016)); especially those with a tendency toward hypertension given the significant negative correlation between baseline BP and training‐induced change in BP observed in this study. Potential mechanisms for this improvement include reduced endothelial dysfunction due to increased nitric oxide bioavailability as well as decreased sympathetic nerve activity, both of which lead to reduced resting arterial pressure (Garg et al. 2014). With no recorded side effects, particularly versus pharmacological intervention, H‐IHGT is a very attractive option to reduce BP given the striking risk reduction in both coronary heart disease events (22%) and stroke (41%) with just 10 mmHg reduction in SBP or 5 mmHg reduction in DBP (Law et al. 2009).

In summary, advancing age, lack of time, climate, and perceived effort are all negatively associated with physical activity participation (Trost et al. 2002). All three of the interventions employed in this study potentially address these issues in that they are time‐efficient, suitable for all ages and can be performed indoors. Indeed, previous studies have also reported HIIT to be more enjoyable and less effortful than traditional endurance exercise for both healthy individuals (Bartlett et al. 2011) and patient groups (Kong et al. 2016). Ongoing debate exists as to the wider public health application of HIIT (Biddle and Batterham 2015), suggesting that, as in this study, low volume or reduced exertion HIIT (RE‐HIIT) may be a more practical and tolerable solution to promote extensive uptake of HIIT, versus the earlier Wingate style HIIT (Gillen and Gibala 2013).

Importantly, all three exercise interventions in this study required a total weekly time commitment of <45 mins. This is 30% less time than the current adult guidelines for vigorous activity and only one‐third of the time commitment recommended for moderate activity (WHO 2015). As a previously identified barrier to exercise, reduction in total time commitment, would likely lead to enhanced exercise adoption and adherence (Trost et al. 2002). Additionally, our findings suggest that the adaptations induced by H‐HIIT and H‐IHGT have potential, particularly as adjuvant home‐based strategies, to improve key aspects of CRF and BP.

We recognize limitations to this study design. The small sample size may increase type II errors, which may mask the potential of L‐HIIT to improve BP given that reductions in BP have previously been shown with L‐HIIT (Boereboom et al. 2016). Equally the improvements in BP noted in the H‐IHGT group may be reflective of regression to the mean and as such larger studies are required to remove this potential error. The intensity and compliance for the home‐based exercise interventions was monitored by self‐report, however, given the improvements in CRF in just 4‐weeks, volunteers in the H‐HIIT group were likely exercising at high‐intensity given the improvements seen despite low total workload, as seen previously (Iaia et al. 2009; Gibala et al. 2012; Gillen and Gibala 2013).

In conclusion, both L‐HIIT and H‐HIIT can safely elicit significant gains in CRF in sedentary middle‐aged individuals in just 4 weeks. Additionally, H‐IHGT can improve BP within the same timeframe with a similar low time commitment. Larger scale studies are required to fully assess the feasibility and effectiveness of these interventions, in healthy and clinical populations, while also exploring the mechanistic basis of adaptation.

## Conflict of Interest

None declared.
